# Band Neutrophils Are Observed in Dogs Undergoing Multiagent Chemotherapy Including Vincristine

**DOI:** 10.3390/ani16030434

**Published:** 2026-01-30

**Authors:** Caitlin N. Eliason, Steven J. Pierce, Alison Masyr

**Affiliations:** 1 Department of Small Animal Clinical Sciences, College of Veterinary Medicine, Michigan State University, East Lansing, MI 48824, USA; eliasonc@msu.edu; 2 Department of Clinical Sciences, College of Veterinary Medicine and Biomedical Sciences, Colorado State University, Fort Collins, CO 80523, USA; 3 Center for Statistical Training and Consulting (CSTAT), Michigan State University, East Lansing, MI 48824, USA; pierces1@msu.edu

**Keywords:** neutrophils, neutrophil band cells, chemotherapy, dogs, vincristine

## Abstract

Chemotherapy is known to cause significant neutropenia, though concomitant left shift (also known as bandemia) has yet to be documented in dogs. The objective of this study is to assess the prevalence of band neutrophil elevation in dogs undergoing multiagent chemotherapy and identify factors influencing bandemia development. Medical records were retrospectively reviewed between 2018 and 2022 for dogs that started multiagent chemotherapy with at least one follow-up visit. Records were analyzed for body surface area (BSA), white blood cell count, segmented neutrophil count, band neutrophil count, and the previously administered chemotherapy drug (vincristine, cyclophosphamide, or doxorubicin). A generalized linear mixed model was used for statistical analysis. Ninety dogs with 530 nadir complete blood counts (CBCs) were identified. Band neutrophils were present in 20.2% of nadir CBCs, and increased band neutrophils occurred in 13.6%. Smaller BSA was associated with higher bandemia prevalence. Bandemia prevalence was 6% higher after vincristine (Risk Difference, 95% CI = [1%, 11%], *p* = 0.026) or doxorubicin (Risk Difference, 95% CI = [−1%, 13%], *p* = 0.115) administration than when patients received cyclophosphamide. Limitations include a lack of standardization of protocol, evaluation of underlying conditions that could contribute to bandemia, and opportunity for laboratory error. This study demonstrates that band neutrophils are present in dogs receiving chemotherapy, with a negative relationship between bandemia and BSA. Bandemia was most common following vincristine and doxorubicin administration.

## Simple Summary

Neutrophils are white blood cells involved in the first line of immune defense. In times of increased neutrophil need, immature band neutrophils are released from the bone marrow. Chemotherapy causes a low neutrophil count, which leads to rapid replenishment, though the presence of band neutrophils in these patients has not been documented in veterinary medicine. This study retrospectively evaluated 90 dogs undergoing multiagent chemotherapy. We evaluated 530 post-chemotherapy complete blood counts for the presence of band neutrophils. We found that band neutrophils were present in 20% of complete blood counts, and increased band neutrophils occurred in 14%. Smaller dogs were more likely to experience increased band neutrophil counts. Band neutrophils were 6% higher after vincristine or doxorubicin chemotherapy administration than when patients received cyclophosphamide. Limitations include a lack of standardization of protocol, evaluation of underlying conditions that could contribute to bandemia, and opportunity for laboratory error. This study demonstrates that band neutrophils are present in dogs receiving chemotherapy, with a negative relationship between band neutrophil count and dog size. Band neutrophils were most common following vincristine and doxorubicin administration.

## 1. Introduction

Hematologic changes during maximum tolerated dose (MTD) chemotherapy are routinely monitored, as myelosuppression may delay further chemotherapy treatment, warrant future treatment alteration, or indicate the need for immediate intervention. The degree of neutropenia that prompts these changes is often arbitrary and at the discretion of the administering clinician [1].

In dogs, neutrophil maturation stages include myeloblasts, promyelocytes, myelocytes, metamyelocytes, band neutrophils, and segmented neutrophils [2]. Neutrophils are believed to require 3–4 days for maturation in the bone marrow from metamyelocytes to segmented neutrophils prior to release into circulation [2]. Segmented neutrophils comprise the majority of white blood cells (WBCs) in circulation and have a half-life of 5–10 h in the blood [2]. Band neutrophils are rarely present in circulation in health. During times of increased neutrophil demand, neutrophil release into the blood from the storage pool in the bone marrow is increased [2]. Concurrently, the rate of maturation of other post-mitotic neutrophil precursors in the bone marrow is accelerated. If severe enough, continued neutrophil demand leads to the release of band cells, termed as left shift or bandemia. Left shift can be further classified as degenerative or regenerative [2]. Degenerative left shift indicates band neutrophils counts in excess of mature neutrophils. This process is commonly seen in inflammatory conditions, including pneumonia, septic peritonitis, gastroenteritis, parvoviral enteritis, and pancreatitis [3]. Regenerative left shift is predominated by mature neutrophils with band neutrophils proportional to the total neutrophilia and consistent with an appropriate response to an inflammatory stimulus [2].

Commonly documented hematologic alterations in veterinary oncology patients receiving chemotherapy include neutropenia, thrombocytopenia, anemia, and increased nucleated red blood cells (nRBCs) [4,5,6]. Increases in nRBC counts are believed to be a response to bone marrow regeneration, compromised bone marrow erythrocyte release, reduced activity of the reticuloendothelial system, or a disrupted blood–bone marrow barrier [5,6]. Neutropenia and thrombocytopenia are believed to reflect the targeted effect of MTD chemotherapy on rapidly dividing cell populations [7,8]. Neutrophil and platelet nadirs typically occur approximately 5–10 days after appropriate chemotherapy dosing [9].

Additionally, the impact of body size and toxicity has been documented in several studies. While the use of body surface area (BSA) is intended to estimate various physiologic processes, small dogs are disproportionately affected by many chemotherapy drugs [10]. Previous studies have demonstrated that dogs with a lower BSA experience more myelosuppression during chemotherapy [11,12]. Other studies have suggested that the BSA formula does not factor in breed specificities in drug metabolism and clearance or the changes in drug metabolism that occur with age or disease [10,13].

Currently, veterinary oncologists utilize standardized metrics for assessing treatment-related adverse events, the Veterinary Cooperative Oncology Group—Common Terminology Criteria for Adverse Events (VCOG-CTCAE) [14]. This includes neutropenia on a scale of grades 1 through 5. Neutropenia grade per VCOG-CTCAE determines an oncologist’s choice to proceed with treatment, delay treatment, begin antibiotic therapy, hospitalize a patient, or even reduce future chemotherapy doses [15,16]. Despite chemotherapy causing profound myelosuppression in some dogs, chemotherapy-induced bandemia has not been reported in veterinary medicine. It stands to reason that bandemia may also be a clinically valuable reference and impact clinician therapeutic choices. To begin evaluating this topic, we chose to focus on dogs receiving multiagent chemotherapy (containing vincristine [VCR], cyclophosphamide [CTX], and doxorubicin [DOX]) as a means of assessing a patient population with lymphoid diseases undergoing a standardized protocol that requires weekly visits. As such, the goal of this study was to retrospectively assess the prevalence of band neutrophil presence and/or elevation (bandemia) in dogs undergoing multiagent chemotherapy and specific factors that influence bandemia development. This article is a revised and expanded version of a poster entitled “Incidence of bandemia in dogs undergoing chemotherapy”, which was presented at the Veterinary Cancer Society Annual Meeting in Orlando, FL, in October 2024 [17].

## 2. Materials and Methods

### 2.1. Data Collection

To best evaluate several MTD drugs across a standardized population, we selected dogs receiving multiagent chemotherapy including VCR, CTX, and DOX for treatment of round cell neoplasia. Medical records of dogs evaluated at the Michigan State University Veterinary Medical Center (MSU VMC) between January 2018 and December 2022 were reviewed. Dogs were included if they received VCR as part of a multiagent chemotherapy protocol and had at least 1 follow-up CBC performed through the Michigan State University Veterinary Diagnostic Laboratory (MSU VDL).

Only the first 10 treatments of each patient’s induction protocol were evaluated for this study, as patients were having weekly chemotherapy and CBCs. CBCs were only included if they were within the expected 5–10-day nadir period, and only one CBC was included per nadir. Patients who had multiple CBCs between treatments only had the 7-day nadir included. MSU VDL CBCs are run on a Siemens ADVIA 2120 Hematology Analyzer (Munich, Germany). All CBCs were submitted to the MSU VDL where all CBCs have a blood smear microscopically reviewed by a trained laboratory professional. Manual WBC differentials are performed if microscopic evaluation is incongruent with automated count, contains band neutrophils, or is otherwise flagged for leukopenia (<1000/µL), leukocytosis (>40,000/µL), lymphocytosis (>15,000/µL), atypical lymphocytes, degenerative left shift, metamyelocytes or earlier precursors, Pelger–Huët cells, and severe neutrophil toxicity.

Data collected from medical records and CBCs included age (years), sex, reproductive status, breed, body surface area (BSA) at the start of chemotherapy (m^2^), total WBC count, segmented neutrophil counts (either automated count or manual), band neutrophil count, and the previously administered chemotherapy drug (VCR, CTX, or DOX) and dosage (mg/m^2^). If band neutrophils were identified, determination of the presence of toxic change was also recorded. Automated neutrophil counts without a manual band neutrophil count were assumed to have no band neutrophils present. Bandemia was defined as any band neutrophil count above the MSU VDL reference interval (RI), the upper limit of which is 0.1 k/µL.

Two measures of sickness were recorded for the subset of observations where bands were present. Degree of sickness was a 4-level, ordinal variable representing the dog’s status at the time the CBC was collected: not sick, self-limited (sick but did not require treatment), medicated (sick and received medication not including antibiotics), or hospitalized (due to sickness). A binary indicator of sickness was also created (0, no; 1, yes).

### 2.2. Statistical Model

Simple statistics (means, medians, standard deviations, interquartile ranges [IQRs], ranges, absolute white blood cell, mature neutrophil, and band neutrophil counts and percentages) were used to describe the raw data, depending on the nature of the variable. The Pearson correlation between total neutrophil count and total band count was estimated, along with a 95% confidence interval. A contingency table summarized the binary indicators of sickness and bandemia in the subset of the data where bands were present and was analyzed with a chi-square test for independence.

The data were clustered: repeated observations (level 1 units) were nested within dogs (level 2 units). A generalized linear mixed model (GLMM) with a logit link function was employed to assess the effects of each drug and BSA on bandemia (0 = absent, 1 = present). We used R 4.5.2 with the glmmTMB 1.1.14 and marginaleffects 0.31.0 packages to fit and interpret the model [18,19,20,21]. A random intercept accounted for variability between dogs. The model included fixed effects for drug (at level 1) and adjusted for mean proportions of exposure to different drugs across observations (at level 2), plus grand-mean-centered BSA. An expanded model used grand-mean-centered age and sex as additional level 2 covariates.

Level 1 drug dummy codes were centered within clusters (i.e., dogs) prior to modeling to ensure unconflated estimates of each predictor’s effect [22]. Interpretation was facilitated by examining estimated marginal means for the drug effect at level 1 and the BSA effect at level 2, the risk difference (RD) for pairwise comparisons of the drugs, and the odds ratio for BSA. We also plotted the marginal predicted bandemia prevalence as a function of BSA.

Multivariate analysis was conducted with the aforementioned variables, setting significance at *p* < 0.05.

### 2.3. Reproducibility

Reproducibility materials for this paper are in the CHOPStudy 1.0.3 package for R (Pierce, 2026), which contains the data, software code used for the analyses, and statistical output [23].

## 3. Results

### 3.1. Patient Population

A total of 342 patients who had received VCR were pulled from the MSU VDL database. Dogs who received VCR for conditions other than round cell neoplasia and feline patients were removed, leaving 144 patients. Dogs receiving VCR as a part of protocols without intent to include CTX and DOX were removed, leaving 90 evaluable patients. The median age at diagnosis was 7 years (mean 7.3 years, SD 2.8 years, IQR 5.0–9.0 years, range 1–14 years). The most commonly represented breeds included mixed breed (*n* = 26); Labrador Retriever (*n* = 6); Golden Retriever, Goldendoodle, Pit Bull Terrier, and Rottweiler (*n* = 5 for each); and Great Dane (*n* = 3) (Table 1).

The median BSA of included patients was 1.00 m^2^ (mean 0.94 m^2^, SD = 0.35 m^2^, IQR 0.67–1.17 m^2^, range 0.26–1.85 m^2^). The median patient weight was 30.85 kg (mean 29.75 kg, SD 15.43 kg, IQR 17.20–39.20 kg, range 4.02–78.20 kg). Twelve patients (13%) weighed less than 10 kg; six patients (7%) weighed between 10 and 15 kg.

The most common diagnoses were multicentric lymphoma (*n* = 62), lymphoma (*n* = 14), and gastrointestinal lymphoma (*n* = 3) (Table 1). World Health Organization (WHO) stage was available in 70 dogs, substage was available in 68 dogs, and immunophenotype was available in 44 dogs.

From these 90 patients, a total of 530 CBCs were evaluated. The number of visits ranged from 1 to 10 with a median of 6.00 treatments (mean 5.89, SD 3.10, IQR 3.00–9.00). All patients included were started on a 25-week multiagent protocol, with the Wisconsin–Madison CHOP protocol (UW-25) [24] being the most common. In total, 64 patients received VCR week 1 followed by CTX week 2, 11 received VCR week 1 followed by a different drug (neither CTX nor DOX) in week 2, and 8 patients received CTX week 1, followed by VCR week 2. One patient received DOX week 1.

### 3.2. CBC Results

#### 3.2.1. Total WBC and Band Neutrophil Count

**Figure 1:**
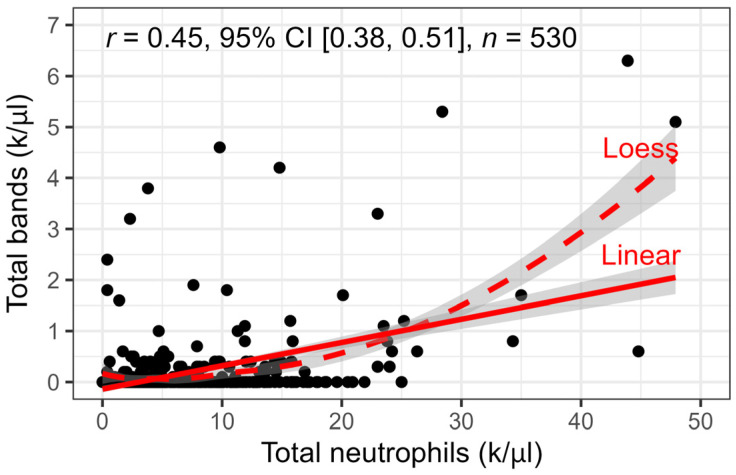
Scatterplot of total bands versus total neutrophils. The variables have a moderate, positive correlation as shown by the linear fit line (solid, corresponding to Pearson correlation) and loess smoothing (dashed) curves with 95% CIs. The loess curve trends upward quickly at higher values of total neutrophils (>k/µL) because the sparse data in that range contains some high-leverage outliers. The outliers affect the linear slope too but not to the same extent.

The median total WBC count was 7.1 k/µL (IQR 4.7–10.9 k/µL, range 0.7–120.2 k/µL; MSU VDL RI 4.6–10.7 k/µL). The median neutrophil count was 4.6 k/µL (IQR 2.6–8.0 k/µL, range 0.0–47.9 k/µL; MSU VDL RI 2.6–7.5 k/µL). Eighty percent (423/530) of CBCs had a band count of 0.0 k/µL. As such, the median number of band neutrophils across all CBCs was 0.0 k/µL (IQR 0.0–0.0 k/µL, range 0.0–6.3 k/µL; MSU VDL RI 0.0–0.1 k/µL). Band neutrophils were present in 20.2% (107/530) of CBCs. Bandemia was noted in 13.6% (72/530) of all CBCs and 67% (72/107) of CBCs with band neutrophils. Of CBCs containing bands, 16% (17/107) had documented toxic change within neutrophils. Band neutrophil count was moderately correlated with neutrophil count (Pearson *r* = 0.45, 95% CI = [0.38, 0.51]) (Figure 1). Only 0.9% (5/530) of CBCs had a band neutrophil count greater than or equal to that of neutrophils.

#### 3.2.2. Sickness and Bandemia

Among the 107 observations where bands were present, the distribution for degree of sickness at the time the CBC was collected showed that in 72.9% (78/107) of the observations, the dog was not sick and in 4.7% (5/107), the dog was sick but this was self-limited and did not require intervention. Among band-containing visits requiring intervention, in 15.0% (16/107), the dog received supportive medications that did not include antibiotics, and in the remaining 7.5% (8/107), the dog was hospitalized. A 2 × 2 contingency table for binary indicators of sickness and bandemia showed that bandemia was present in 67% (72/107) of the observations where bands were present but the dog was sick in only 27% (29/107) of them. Bandemia was detected in 62.8% (49/78) of the observations where the dog was not sick and 79.3% (23/29) of the observations where the dog was sick. A chi-square test for independence failed to detect an association between sickness and bandemia among band-containing visits, χ^2^ = 1.92, *p* = 0.166.

#### 3.2.3. Band Neutrophil Count Following Chemotherapy Administration

VCR was administered 292 times, CTX was administered 134 times, and DOX was administered 104 times (Table 2). Bandemia was most frequently observed following VCR and DOX administration. Regardless of drug administered, when band neutrophils were present, there were sufficient numbers to be considered bandemia in 58–76% of cases.

### 3.3. Multivariate Analysis

Table 3 shows the parameters and fit statistics for the employed model. Adding age and sex as additional covariates did not improve the model, χ^2^(2) = 0.409, *p* = 0.815, so we report the model without them. The adjusted intraclass correlation shows that 31% (95% CI = [5%, 50%]) of unexplained variance in bandemia is attributable to individual differences between patients. The marginal R^2^ shows that fixed effects explain 10% (95% CI = [4%, 26%]) of the variance in bandemia and the conditional R^2^ shows that the fixed and random effects together explain 38% (95% CI = [19%, 57%]) of the variance [25].

**Figure 2:**
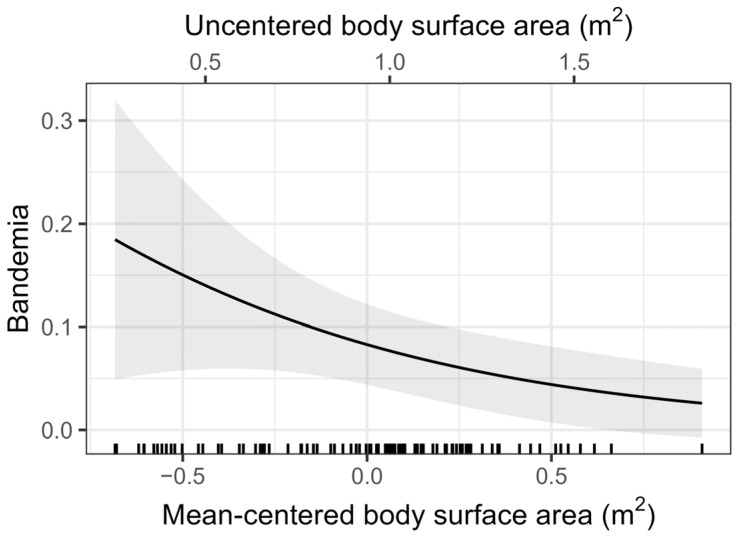
Body surface area is negatively associated with bandemia: prevalence decreased as BSA increased (OR = 0.261, 95% OR CI = [0.076, 0.897], *p* = 0.033). Predicted prevalence is about 19% for the smallest dog, 8% for average-size dogs (centered mean BSA = 0.00 m^2^, mean BSA = 0.94 m^2^), and 2.5% for the largest dog.

The model revealed a negative association between BSA and bandemia (Figure 2) such that larger patients had a lower prevalence of bandemia than smaller patients (OR = 0.261, 95% OR CI = [0.076, 0.897], *p* = 0.033). Notably, mean exposure to different drugs across observations did not exhibit noteworthy effects within each dog.

**Figure 3:**
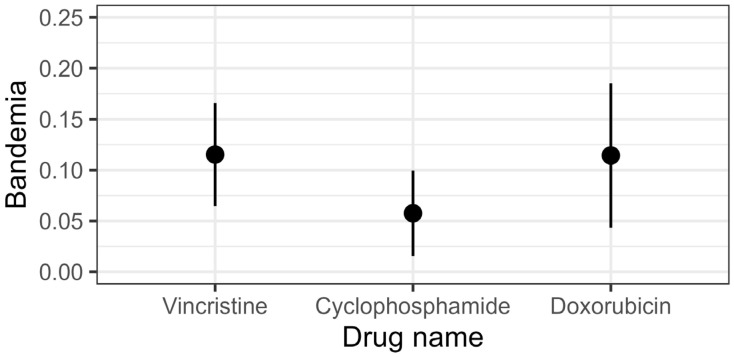
Estimated marginal means for bandemia prevalence by drug. Bandemia prevalence was 6% higher when vincristine (RD = 0.06, 95% CI = [0.01, 0.11], *p* = 0.029) or doxorubicin (RD = 0.06, 95% CI = [−0.02, 0.13], *p* = 0.123) was administered than when patients received cyclophosphamide.

Estimated marginal means from the GLMM (Figure 3) showed that when chemotherapy drugs were considered individually, VCR administration was associated with a bandemia prevalence of 12% (95% CI = [6%, 17%]), CTX with an prevalence of 6% (95% CI = [2%, 10%]), and DOX with an prevalence of 11% (95% CI = [4%, 19%]). Pairwise comparisons showed that risk of bandemia was 6% higher (RD, 95% CI = [1%, 11%]) following VCR than with CTX (*p* = 0.029). While the risk of bandemia after DOX was also 6% higher (RD, 95% CI = [−2%, 13%]) than for CTX, the wider confidence interval overlapped zero, so it is plausible that there was no difference (*p* = 0.123). There was little evidence for any difference between VCR and DOX administration with regards to bandemia prevalence (RD = 0%, 95% CI = [−7%, 7%], *p* = 0.980).

## 4. Discussion

In the present study, we evaluated the prevalence of band neutrophils and bandemia in dogs undergoing VCR, CTX, and DOX multiagent chemotherapy. Results of this retrospective study demonstrate that band neutrophils or bandemia can be present in dogs receiving chemotherapy. To our knowledge, this is the first documentation of band neutrophils and bandemia in dogs. For dogs included in this study, band neutrophils and bandemia were present in 20.2% and 13.6% of cases, respectively. Notably, of the CBCs with band neutrophils present, 67% had band neutrophil counts above the RI.

Bandemia was more frequently observed at timepoints following VCR, and possibly also DOX administration, as compared to CTX administration. CTX has been suggested to be less cytotoxic to stem cells than other chemotherapy drugs [26,27,28,29] and is less likely to result in sepsis [11], which could explain fewer cases experiencing bandemia. During the course of its metabolism, CTX is inactivated via aldehyde dehydrogenase (ALDH) [30]. Lymphoid cells are susceptible to the cytotoxic effects of CTX given their low ALDH expression; however, stem cells show much higher ALDH expression, giving them greater resistance to the drug [31]. As such, CTX is frequently used to lymphodeplete oncology patients prior to stem cell collection for transplant [29,32,33]. These patients receive much higher doses (500–650 mg/m^2^) than those used in multiagent chemotherapy (200–250 mg/m^2^) [8,24], though only 26–40% of dogs experience grade 4 neutropenia [32,33].

Based purely on assessing *p*-values, bandemia was not significantly more likely to be observed at timepoints following DOX as compared to CTX administration. Since DOX is typically administered at the fourth week of UW-25, this drug was administered the least number of times. The decreased amount of data points resulted in a wider confidence interval, which barely overlaps with zero. We suspect that the lack of statistical significance may be the result of a type II error as nearly all plausible values for the effect are positive and the point estimate for the RD is the same as that between VCR and CTX.

Given that previous studies have shown that patients with a lower BSA are more likely to experience side effects of chemotherapy [10,12,34], we expected to see a similar trend with bandemia. This negative association between BSA and bandemia development was observed. Small dogs receive a higher dose per body weight when prescribed based on BSA. For this reason, many clinicians dose DOX based on body weight (kg) for dogs under 10–15 kg while using BSA (m^2^) dosing for larger dogs [13].

While this study has documented that band neutrophils are present during chemotherapy, both within and above RIs, further study is warranted to determine the impact on therapy for dogs. Neutropenia has previously been indicated as a positive prognostic factor in dogs with lymphoma receiving chemotherapy, but these studies did not evaluate the effect or presence of band neutrophils [7,26]. The presence of band neutrophils in the context of a degenerative left shift was described as a negative prognostic indicator in dogs in one study, though this was not limited to cancer patients and did not include drug-induced myelosuppression [3]. In human cancer patients with advanced uterine cervical cancer, pre-treatment bandemia has been identified as a negative prognostic indicator [35]. Pre- and post-amputation band neutrophil counts were recently shown to serve as independent prognostic factors for canine osteosarcoma patients [36]. This may suggest that band neutrophil count serves as a biomarker of systemic immune status, especially given that immature neutrophils often fall into the category of myeloid-derived suppressor cells. This prognostic association has yet to be defined in human or canine lymphoma patients.

The multi-level model employed indicated that 31% of unexplained variance was the result of individual dog characteristics. Certain dogs tended to consistently have elevated band neutrophils counts, while others did not consistently demonstrate this finding. BSA may be included in these individual characteristics, though there are likely others. The intention of this study was to determine the prevalence of bandemia in dogs and begin to consider factors influencing this finding. When including fixed effects (including chemotherapy drug and BSA) along with individual characteristics, the model could explain 38% of the total variance seen in bandemia. It is possible that straightforward factors, like breed, reproductive status, or lifestyle habits may play a role in bandemia development, but our results did not support associations with age or sex. Future studies should also consider lymphoma immunophenotype, disease distribution (including stage and substage, when applicable), and genomic and cytokine profiles. Furthermore, while the cumulative exposure to each drug did not appear to affect bandemia occurrence, future investigations may also consider the presence of a temporal influence on bandemia development as patients progress through prolonged protocols. Given the patients evaluated received a multiagent protocol, this study cannot account for the confounding influence of the drugs on each other. The authors are interested in engaging in future, prospective studies monitoring dogs receiving single-agent chemotherapy protocols. This type of study design would facilitate a more uniform study population, treatment protocol, and data collection, including outcome data.

The aim of this study was to document the frequency of a clinical observation and provide a foundation for future studies on the topic of bandemia in dogs. The present study has several limitations, which are predominantly linked to its retrospective nature. Underlying inflammatory conditions, including concurrent illness, infection, or stress were not assessed and therefore cannot be ruled out as causes for bandemia. We recorded clinical wellbeing in instances of band neutrophils and found that in 77.6% of band-containing CBCs, dogs were not ill or were mildly unwell with self-resolving signs. This suggests that the presence of band neutrophils in nadir CBCs is not inherently a cause for alarm. In this subset of our population, less than 8% of CBCs with bands belonged to dogs that required hospitalization. This is notably lower than the rate of hospitalization identified in a recent study of dogs undergoing chemotherapy treatment, which was approximately 24% [37].

In addition, patients were treated by several clinicians and therefore may have received varied drug doses. The treatment schedule was not standardized. While the UW-25 protocol is the most common CHOP schedule followed at MSU VMC, the treatment order was changed according to individual patient needs and comorbidities. It was not documented which patients were on steroids, at what dose, and when they were tapered. Furthermore, this is a single-institution study and all CBCs were performed with MSU VDL analyzers. While all CBCs are evaluated by an MSU VDL technologist and/or a board-certified clinical pathologist, differentiation between the mature and immature stages is subjective and prone to human or lab error [38]. Rare blood cell conformations, like those in Pelger–Huët anomaly, could not be ruled out, though only two patients in this study were listed as Australian Shepherd dogs [39]. Finally, neutrophil nadirs are most often measured at 7 days, but a range of 5–10 days has been suggested, meaning some of our measured CBCs could be from before an individual’s true nadir or after the nadir had started to resolve [16,40].

## 5. Conclusions

In conclusion, this study is the first of its kind to describe band neutrophils in dogs receiving multiagent chemotherapy. Band neutrophils were most frequently observed at timepoints following VCR and DOX administration as compared to CTX treatment. Dogs with smaller BSA were more likely to develop bandemia following chemotherapy administration. Given that bandemia was observed in 1 of every 7 CBCs, future studies are needed to determine causal influences on bandemia development and any therapeutic impact of bandemia in dogs undergoing chemotherapy.

## Figures and Tables

**Table 1:** Summary of patient characteristics, diagnosis, disease staging, and immunophenotype.

Characteristic	N (%)
Breed	
Mixed breed	26 (29)
Labrador Retriever	6 (7)
Golden Retriever	5 (6)
Goldendoodle	5 (6)
Pit Bull Terrier	5 (6)
Rottweiler	5 (6)
Great Dane	3 (3)
Australian Shepherd	2 (2)
German Shepherd	2 (2)
Greyhound	2 (2)
Pug	2 (2)
Shih Tzu	2 (2)
Siberian Husky	2 (2)
Other *	23 (26)
Reproductive status	
Neutered male	48 (53)
Spayed female	34 (38)
Intact male	7 (8)
Intact female	1 (1)
Diagnosis	
Multicentric lymphoma	62 (69)
Lymphoma	14 (16)
Gastrointestinal lymphoma	3 (3)
Mediastinal lymphoma	2 (2)
Splenic lymphoma	2 (2)
Other **	7 (8)
Multicentric lymphoma stage	
1	1 (1)
2	0 (0)
3	29 (32)
4	15 (17)
5	25 (28)
Not available	20 (22)
Multicentric lymphoma substage	
A	28 (31)
B	40 (44)
Not available	22 (24)
Immunophenotype	
B	29 (32)
T	15 (17)
Not available	46 (51)

* Consisted of one dog each of American Cocker Spaniel, Beagle, Bernese Mountain Dog, Border Collie, Boxer, Bullmastiff, Chesapeake Bay Retriever, Chihuahua, Cavalier King Charles Spaniel, Coonhound, Dogo Argentino, English Bulldog, English Cocker Spaniel, English Setter, English Springer Spaniel, French Bulldog, Jack Russel Terrier, Miniature Poodle, Miniature Schnauzer, Portuguese Water Dog, Shetland Sheepdog, Welsh Terrier, and West Highland White Terrier. ** Consisted of 1 each of acute myeloid leukemia, chronic lymphocytic leukemia, cutaneous lymphoma, hepatosplenic lymphoma, large granular lymphoma, lymphoproliferative neoplasm, and renal lymphoma.

**Table 2:** Summary of band neutrophil counts following chemotherapy administration.

Drug	VCR	CTX	DOX
Median dosage (mg/m^2^) (IQR, range)	0.6 (0.6–0.7, 0.5–0.7)	250.0 (240.0–250.0, 180.0–300.0)	30.0 (25.0–30.0, 16.0–31.0)
Median band neutrophil count (k/µL)	0.0	0.0	0.0
IQR band neutrophil count (k/µL)	0.0–0.0	0.0–0.0	0.0–0.0
Range band neutrophil count (k/µL)	0.0–6.3	0.0–5.3	0.0–1.1
Band neutrophil count within RI (n, %)	22/292, 7.5	8/134, 6.0	5/104, 4.8
Bandemia (n, %)	45/292, 15.4	11/134, 8.2	16/104, 15.4
Bandemia in CBCs with band neutrophils (n, %)	45/67, 67	11/19, 58	16/21, 76

**Table 3:** Logistic generalized linear mixed model predicting presence of bandemia.

Term	Estimate [95% CI]	Odds Ratio [95% CI]	z Score	*p* Value
Level 1 fixed effects				
Intercept	−2.489 [−4.281, −0.696]	0.083 [0.014, 0.499]	−2.721	0.007
Drug: CTX	−0.984 [−1.766, −0.202]	0.34 [0.171, 0.817]	−2.465	0.014
Drug: DOX	0.164 [−0.540, 0.869]	1.179 [0.583, 2.385]	0.457	0.647
Level 2 fixed effects				
Mean CTX exposure	2.113 [−2.231, 6.456]	8.271 [0.107, 636.722]	0.953	0.340
Mean DOX exposure	−2.289 [−6.875, 2.296]	0.101 [0.001, 9.938]	−0.978	0.328
BSA (m^2^)	−1.344 [−2.579, −0.109]	0.261 [0.076, 0.897]	−2.133	0.033
Level 2 random effect				
Patient intercept (SD)	1.220 [0.798, 1.865]			

Note: Model fit via maximum likelihood estimation to data from 90 dogs and 530 observations. Level 1 drug dummy codes were centered within patients, and body surface area was grand-mean-centered. Drug reference level was vincristine. Model df = 7. Residual df = 523. AIC = 397.5. BIC = 427.4. RMSE = 0.29. Log likelihood = −191.753. Deviance = 383.505. CTX = cyclophosphamide. DOX = doxorubicin.

## Data Availability

The original data presented in the study are openly available in a GitHub repository at https://github.com/sjpierce/CHOPStudy (accessed on 27 January 2026).

## Abbreviations

The following abbreviations are used in this manuscript:

BSA	Body Surface Area
CBC	Complete Blood Count
CTX	Cyclophosphamide
DOX	Doxorubicin
GLMM	Generalized Linear Mixed Model
MSU VDL	Michigan State University Veterinary Diagnostic Laboratory
MSU VMC	Michigan State University Veterinary Medical Center
MTD	Maximally Tolerated Dose
RD	Risk Difference
RI	Reference Interval
VCR	Vincristine
WBC	White Blood Cell
